# Strategies for reducing the time of mechanical ventilation and ventilator-associated pneumonia

**DOI:** 10.1186/cc10192

**Published:** 2011-06-22

**Authors:** BLDS Guimaraes, SN Nemer, LM Azeredo, JB Caldeira, GM Souza, F Rodriguez, E Guimarães, LPPCPSM Damasceno

**Affiliations:** 1 Hospital de Clínicas de Niterói, Niterói - RJ, Brazil

## Introduction

Ventilator-associated pneumonia (VAP) is one of the most frequent causes of nosocomial infection and complication in the intensive care unit (ICU). VAP is associated with increased mortality and morbidity, as well as increased costs of intensive therapy.

## Objective

To compare the prevalence of VAP and the duration of mechanical ventilation in a general ICU, before and after implantation of a bundle of four and five measures.

## Methods

A prospective study made in the general ICU, from December 2007 to November 2009, with a total of 432 patients. The measures adopted in the bundle of VAP were: daily sedative interruption, elevation of the head of the bed to 45°, deep venous thrombosis prophylaxis, peptic ulcer disease prophylaxis. The fifth measure used was the daily interruption of sedatives with spontaneous breathing trials (SBTs). The control group was the group without the VAP bundle. Group 1 was with the VAP bundle. Group 2 was the group of VAP bundle with daily interruption of sedatives and SBTs.

## Results

**Figure 1:**
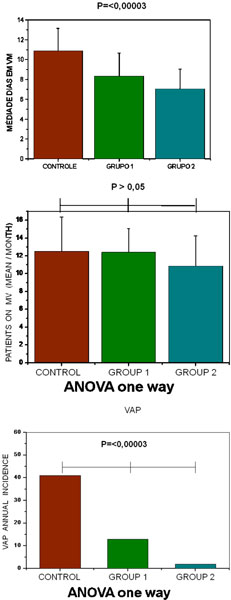


Control group: 153 patients were ventilated from December 2006 to November 2007, with a mean ventilation time of 10.8 ± 2.2 days, as 41 patients were with VAP, 27.3% of VAP with 53.3% mortality. Group 1: 149 patients were ventilated from December 2007 to November 2008, with a mean ventilation time of 8.3 ± 2.3 days, as 13 patients were with VAP, 8.7% of VAP with 42% mortality. Group 2: 130 patients were ventilated from December 2008 to November 2009, with a mean ventilation time of 7 ± 2 days, as two patients were with VAP, 1.5% of VAP with 41.5% mortality. All VAP cases on 15 patients happened after the fourth day of MV; that is, all of them were cases of late VAP. See Tables 1 and 2 and Figure 1.

**Table 1:** 

	Control group	Group 1	Group 2
% VAP	27.3	8.7	1.5
% VAP/1,000 days on MV	25.7	10.6	2.2
% death	63.3	42.9	41.5

**Table 2:** 

	Control group	Group 1	Group 2
Gender (men/women)	77/76	82/67	64/66
Age (years)	63.4 ± 19.3	70.7 ± 14.5	66 ± 15.1
APACHE II	17.3 ± 7	15.9 ± 13.3	25 ± 8.8
Diagnosis on admission to intensive care, *n *(%)			
Stroke	15 (9.8%)	4 (2.6%)	3 (2.3%)
SDRA	2 (1.3%)	0 (0%)	0 (0%)
Cardiorespiratory arrest	5 (3.2%)	9 (6%)	7 (5.3%)
Sepsis	25 (16.3%)	15 (10%)	12 (9.2%)
Pneumonia	24 (15.6%)	28 (18.7%)	22 (16.9%)
COPD	12 (7.8%)	16 (10.7%)	16 (12.3%)
Postoperative abdominal surgery	31 (20.2%)	30 (20.1%)	30 (23%)
Oncologic	13 (8.4%)	18 (12%)	14 (10.7%)
Miscellaneous	26 (16.9%)	29 (19.4%)	26 (20%)
Total	153	149	130

## Conclusion

Implementation of a daily bundle with SBTs is associated with reduction of mechanical ventilation time, and it is the determinant factor to have lower indexes of VAP.

